# Clinical Lectures on Diseases of the Ear

**Published:** 1873-06

**Authors:** E. L. Holmes

**Affiliations:** Professor of Ophthalmology and Otology, Rush Medical College; Surgeon to Illinois Charitable Eye and Ear Infirmary, Chicago


A MONTHLY RECORD OF

*Medicine, Surgery and the Collateral Sciences.*

EDITED BY J. ADAMS ALLEN, M.D., LL.D.; AND WALTER HAY, M.D.

VOL. XXX. — JUNE, 1873. — NO. 6.

### Original Communications.

ARTICLE I.—*Clinical Lectures on Diseases of the Ear.* By E. L. HOLMES, M.D., Professor of Ophthalmology and Otology, Rush Medical College; Surgeon to Illinois Charitable Eye and Ear Infirmary, Chicago.

### AURAL CATARRH.

*Gentlemen*—Catarrhal inflammation of the middle ear, in its acute or its chronic form, is a serious and obstinate affection. You must have become impressed with this fact both from your own reading and observation at these clinics. Remember that this class of diseases is a special illustration of the general fact that nearly all mucous membranes, once affected with catarrhal inflammation, are rendered susceptible, and consequently liable, to relapse.

The disease is, in popular language, "nothing but a cold in the ears." To those of you who have seen so many affections of the eye, throat, lungs and other organs, produced by colds, these words "nothing but" must seem wholly misapplied.

Recalling to your minds the mechanism of hearing, you can readily comprehend how inflammation of the mucous membrane of the middle ear, either acute or chronic, by producing thickening of this tissue, must retard the vibratory motions of the membrana tympani, ossicles, especially the stapes, and of the membrane of the foramen rotundum.

The immediate cause of this affection is generally exposure to changes of temperature. The inducing causes may be the susceptibility produced by diseases of the throat, measles, scarlet fever, and, less frequently perhaps, by syphilis.

The disease is often attended with trouble of the throat and nares. The mucous membrane of the upper air passages should always be carefully investigated in every patient with affections of the middle ear.

The symptoms of chronic aural catarrh are usually more or less gradual, sometimes sudden but not extensive diminution in the acuteness of hearing, *tinnitus aurium*, more or less thickening and opacity of the membrana tympani. This membrane is often more concave and irregular in its surface than in the normal condition. The vessels near the handle of the hammer are frequently congested. In some cases, collections of mucus may be detected, in examining the membrane, by the presence of a dark appearance in a portion of the membrane, which is analogous to hypopion in the eye. It is important for you to study the use of the otoscope in detecting the presence and the peculiar qualities of the sounds produced by forcing air through the eustachian tubes into the middle ear, in making strong efforts to blow the nose when the nostrils are closed.

The symptoms of the acute form are, pain, sudden and great loss of hearing, and great redness of the membrana tympani, often extending to the walls of the external meatus. The accumulations of mucus may so far fill the middle ear as to produce a convex appearance of the membrana.

The pain is usually severe, and is accompanied with throbbing and with subjective sounds.

Occasionally cases will be presented in which a "cold" has apparently produced a loss of hearing greater than the other symptoms may seem to indicate.

Inflammation of the middle ear is quite common in young children. As general practitioners you should acquire the habit of never leaving a crying infant without examining the ears. Careful and experienced physicians have for days overlooked the presence of serious disease of the ear in infants evidently in great pain.

In considering the differential diagnosis between catarrhal deafness and deafness from disease of the auditory nerve, you should not forget to study the use of the tuning fork. If neither this instrument nor a watch can be heard a short distance from the ear, but can be heard as readily as in health, when placed in contact with the head or teeth, you can be reasonably certain that the disease is confined to the middle ear, or rather perhaps to the vibratory apparatus, external to the labyrinth.

In regard to the prognosis, I can only say you should be guarded in expressing your opinion, for the ear once having suffered from a catarrhal inflammation, is liable to suffer relapse on exposure to cold. A very large proportion of the cases you will meet, will probably remain with hearing more or less impaired.

In the treatment of the acute forms, where there are severe pain and redness, there is need of promptness and energy.

A few leeches should be applied to the mouth of the meatus, or in front of it. Water, as warm as can be well borne, turned frequently into the meatus, will greatly aid in relieving pain, as also hot compresses applied on and around the ear. Hypodermic injections of morphine may be necessary to secure rest. If you can control the patient, as regards exposure to cold, let him take frequently either hot or Turkish baths, and make free use of diuretics.

As soon as collections of mucus or pus are observed pressing the membrana tympani outward, you should at once perforate this membrane, to ensure their evacuation. The collections must be further removed, either by the use of Politzer's air bag, now so often employed, or by carefully forcing some mild astringent solution into the external meatus through the opening in the membrana tympani and through the eustachian tube.

The syringe should be provided with a nozzle of soft rubber, large enough to close the mouth of the external meatus.

In the chronic and sub-acute forms of the disease, our attention must be carefully directed to the treatment of the mucous membrane of the throat, nares, and eustachian tubes.

The use of gargles, injections of solutions and vapors, are of special importance. These are usually medicated with alum, zinc, tannin, chlorate of potash, muriate of ammonia, or iodine. A few drops of a strong solution of nitrate of silver, thrown just against the mouth of the eustachian tube, where the mucous membrane of this passage is especially implicated, is sometimes of service. This and other remedies must be introduced very carefully by means of the eustachian catheter. The various uses of this instrument should be studied by you all. While you are pursuing practical anatomy, do not omit to observe, repeatedly, the relative position of the opening of the eustachian tubes and posterior nares. Practice your hands in the gentle introduction of the catheter into these tubes. The use of Politzer's air bag, which you have so frequently seen, should especially claim your attention.

I have sketched a mere outline of the treatment of the disease under consideration. If you have an opportunity of attending the Eye clinics of the large cities of this country, or of Europe, you will be convinced, I think, that our most approved treatment is competent to give permanent and radical relief in but a small minority of cases.

Do not, however, neglect nor despise the means we possess, for any treatment that will accomplish so much to relieve a great physical infirmity, with its attending mental suffering, is worthy of careful study.

